# Head and Neck Pathology and Radiology 2011

**DOI:** 10.1155/2012/320319

**Published:** 2012-12-31

**Authors:** Neil S. Norton, Preetha P. Kanjirath, Pilar Hita-Iglesias, Paul C. Edwards

**Affiliations:** ^1^Department of Oral Biology, School of Dentistry, Creighton University, Omaha, NE 68178, USA; ^2^Preclinical Program, College of Dental Medicine-Illinois, Midwestern University, 555-31st Street, 102 Redwood Hall, Downers Grove, IL 60515, USA; ^3^Department of Periodontics and Oral Medicine, School of Dentistry, University of Michigan, Ann Arbor, MI 48109, USA; ^4^Oral and Maxillofacial Surgery, School of Dentistry, University of Michigan, Ann Arbor, MI 48109, USA

Beyond an examination of the dentition and surrounding periodontal tissues, one of the most important tasks of the dental clinician is the accurate diagnosis and management of patients with nontooth-related conditions of the head and neck. For patients with these conditions, it is imperative that the dental clinician performs a comprehensive evaluation and assessment on patients to increase the potential for a successful outcome. The skills of a myriad of specialists will be involved, including those in oral and maxillofacial pathology, radiology, oral medicine, head and neck anatomy, and the primary care provider.

In this special issue dedicated to head and neck pathology and radiology, the manuscripts selected for publication further demonstrate the significance of the relationship described between the specialists and primary care provider. The article by D. C. Lorenzoni et al. highlights radiology and discusses the radiation doses that are associated with plain radiographs, cone beam computed tomography (CBCT), and conventional computed tomography (CT), placing a special emphasis on orthodontics. A. Sharma and V. P. Singh thoroughly summarize a study detecting supernumerary teeth in Indian children in 300 cases using clinical and radiographic examination. In one of their two articles in this special issue, L. Feller and J. Lemmer provide an extensive review on oral leukoplakia and its relationship to human papillomavirus (HPV). P. Bhirangi et al. elaborate on the technical steps for the prosthetic rehabilitation of an edentulous glossectomy patient. In the other article, L. Feller and J. Lemmer critically review cell transformation and the evolution of a field of precancerization as it relates to oral leukoplakia.

